# Diagnosis of a Gastrointestinal Stromal Tumor With Spontaneous Rupture

**DOI:** 10.7759/cureus.35872

**Published:** 2023-03-07

**Authors:** Ryan Meader, Assad Munis, Dean Silas

**Affiliations:** 1 Internal Medicine, Advocate Lutheran General Hospital, Park Ridge, USA; 2 Gastroenterology, Advocate Lutheran General Hospital, Park Ridge, USA

**Keywords:** gastrointestinal stromal tumors, ct abdomen and pelvis with iv contrast, spontaneous rupture, hemorrhage, ct angiogram, spontaneous hemoperitoneum

## Abstract

Gastrointestinal stromal tumors (GIST) are uncommon tumors accounting for 1% of gastrointestinal neoplasms. The most common location of GISTs is in the stomach. Commonly, these tumors present incidentally with an increased presence within older patients. Spontaneous rupture of a GIST is a rare presentation of this uncommon tumor. Our case highlights the diagnostic dilemma and imaging that helped diagnose an abnormal presentation of a ruptured GIST in a young patient.

## Introduction

Gastrointestinal stromal tumors (GIST) are uncommon tumors accounting for 1% of gastrointestinal neoplasms [1]. These tumors commonly present incidentally and, if symptomatic, usually present with non-specific symptoms of nausea, vomiting, abdominal distention, and pain [1]. The median age of GIST presentation is 65 years old [1]. GIST rarely presents with rupture and intra-abdominal hemorrhage [1]. Here, we report the case of a young female who presented with abdominal pain and a spontaneous perforated gastric GIST causing hemoperitoneum.

## Case presentation

A 37-year-old female with no past medical history presented to the emergency department (ED) with two weeks of worsening postprandial left upper abdominal pain. She was in minimal distress, had a heart rate of 119 beats per minute, and was normotensive. There was no history of abdominal trauma. Physical examination revealed mild tenderness in the left upper quadrant, but otherwise a flat abdomen with normal bowel sounds, no guarding, rebound tenderness, or palpable masses. Laboratory studies showed a hemoglobin of 11.4 g/dL (13-17 g/dL), normal liver function tests, and normal lipase levels. Computed tomography (CT) of the abdomen and pelvis with contrast revealed a large irregular heterogeneous fluid collection along the posterior of the gastric body. She received intravenous (IV) fluids and a proton pump inhibitor. Fourteen hours after the initial presentation, her abdominal examination remained unchanged, but tachycardia persisted with a drop in hemoglobin to 8.8 g/dL without clinical evidence of gastrointestinal bleeding. A CT angiography was then performed that revealed a larger heterogeneous fluid collection in the lesser sac, intimately associated with the stomach, and increased fluid in the peritoneum (Figure 2). The findings were suggestive of hemoperitoneum, and the patient was taken for emergent laparotomy which revealed a perforated mass (Figures 3, 4). She underwent a subtotal gastric resection with gastrojejunostomy. Upon pathological examination, confirmation of a GIST was made when the tumor cells were diffusely positive for CD117. She had an uneventful postoperative course and was discharged home in four days.

**Figure 1:**
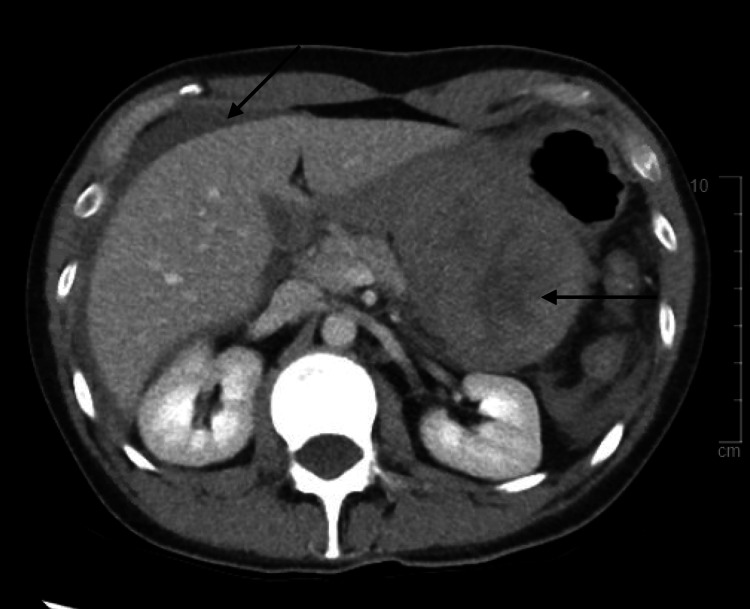
CT of the abdomen and pelvis with contrast performed at presentation showing an irregular large fluid collection upon the gastric body with a small volume of dense fluid.

**Figure 2:**
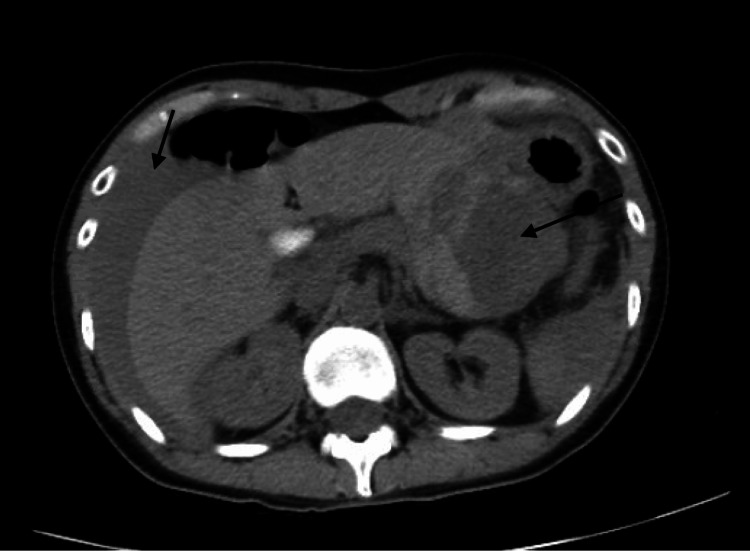
CT angiography performed 14 hours after the initial presentation showing a mass intimately associated with the stomach with associated hypervascularity consistent with a GIST. There is an increased amount of fluid within the abdomen concerning for hemoperitoneum. GIST = gastrointestinal stromal tumor

**Figure 3:**
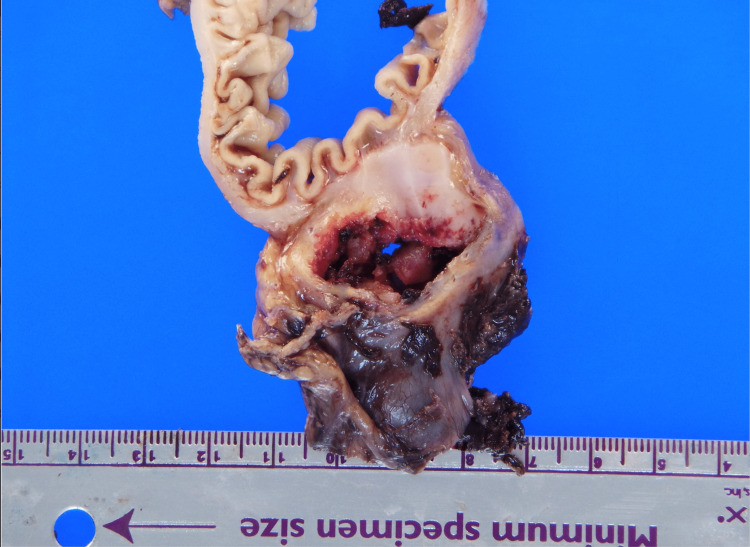
Gross specimen of the perforated GIST. GIST = gastrointestinal stromal tumor

**Figure 4:**
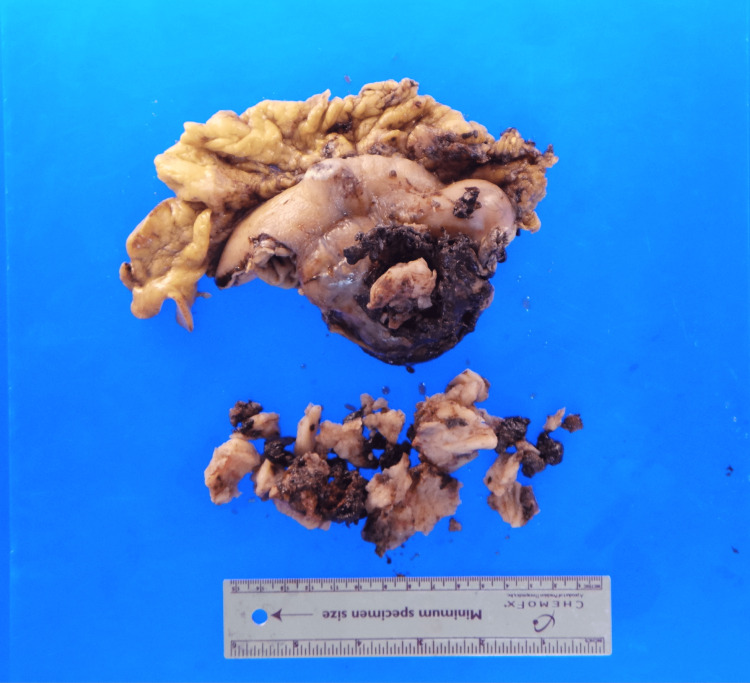
Another gross specimen of the perforated GIST. GIST = gastrointestinal stromal tumor

## Discussion

GISTs are neoplasms that are believed to originate from the interstitial cells of Cajal or related stem cells [1]. GIST tumor locations are 56% in the stomach, 32% in the small bowel, 6% in the colon and rectum, 0.7% in the esophagus, and 5.5% in other locations [1]. These tumors were discovered to have a gain of function mutation that expresses CD117 antigen (C-Kit) [2]. This mutation is generally responsible for activating these tumors and promoting their growth [2]. GISTs that occur outside the stomach are associated with a higher malignant potential, with about 10-30% of GISTs having aggressive behavior [1].

The median age of presentation of GISTs is 65 years old and presents with an even gender distribution [1,3]. GISTs rarely present in patients under the age of 40 [4]. In 18% of patients, GISTs will present asymptomatically [3,5]. About 15 cases previously reported in the literature including this report have described a presentation of a spontaneously ruptured gastric GIST [6]. Asymptomatic tumors can be found incidentally in various ways such as through abdominal CT scans, endoscopy, or during other surgical procedures [4]. Symptomatic patients can present with various gastrointestinal symptoms such as nausea, vomiting, abdominal distention, and abdominal pain [5]. GIST can vary in size with larger tumors causing nearby obstruction of organs leading to dysphagia, obstructive jaundice, or constipation [1,5]. If the neoplasm has perforated, the presentation will show signs of peritonitis or gastrointestinal bleeding [5].

The diagnostic modalities of ultrasound, CT scan, magnetic resonance imaging (MRI), and positron emission tomography (PET) have been discussed to help identify GISTs [1,7,8]. Ultrasound is not useful unless the tumor was larger than 5 cm [1,7,8]. CT enterography was shown to be superior to CT, MRI, and PET [1,7,8]. A CT-guided biopsy also has a role in definitively diagnosing GISTs [1,7,8]. The use of endoscopy was studied and was found to have a limited role in GIST diagnosis because there is a high prevalence of extraluminal tumors [1,7,8]. MRI takes longer and may not be efficient in quickly diagnosing a perforated GIST [1,7,8]. CT angiography was not compared in this review. In our case, CT of the abdomen and pelvis with contrast was inconclusive in diagnosing the patient’s GIST. CT angiography was quickly performed which helped more definitively diagnose the lesion and aided prompt recognition to help take the patient for emergent laparotomy.

GIST treatment typically starts with surgical resection through laparoscopy or laparotomy as the preferred method of treatment, with laparotomy being preferred if the patient is unstable [9]. Tumor rupture increases the risk of tumor metastases and recurrence which occurred in our patient [10]. As far as medical therapy is concerned, approved medications include imatinib, sunitinib, and ponatinib, which are tyrosine kinase receptor inhibitors [10,11].

## Conclusions

The spectrum of clinical manifestations can make the diagnosis of a GIST a clinical dilemma. GISTs, which typically are asymptomatic, are usually found incidentally in older patients. A spontaneous rupture of a GIST is seldom reported in the literature. Initial CT of the abdomen and pelvis with contrast cannot clearly identify the lesion. The free fluid in the patient’s abdomen on initial imaging, tachycardia, and acute drop in hemoglobin raised concern for potential hemoperitoneum from ruptured GIST, which was not confirmed until a CT angiography was performed. This case illustrates the value of CT angiography in visualizing and effectively confirming active intra-abdominal hemorrhage in the setting of a ruptured GIST. This imaging modality allowed for proper recognition of the disease and for our patient to be taken for emergent laparotomy. This case draws importance to the recognition of a prompt diagnosis with imaging. This is a testament to the principle of forming an extensive differential diagnosis to avoid delayed treatment.
